# Waistline to thigh circumference ratio as a predictor of MAFLD: a health care worker study with 2-year follow-up

**DOI:** 10.1186/s12876-024-03229-4

**Published:** 2024-04-24

**Authors:** Xiaoyan Hao, Honghai He, Liyuan Tao, Wei Zhao, Peng Wang

**Affiliations:** Medical examination center, Peking University, Third Hospital, North Garden Road & 49, Beijing, China

**Keywords:** Health check, Cox proportional hazard regression, Functional community, Body composition analysis, Risk, Uneven fat distribution, Clinical characteristics, Subgroup analysis, Incident, Occupational groups, Ultrasonography

## Abstract

**Background:**

This study aimed to determine whether the waist-to-thigh ratio (WTTR) is associated with the incidence of metabolic-associated fatty liver disease (MAFLD) in health care workers.

**Methods:**

There were 4517 health care workers with baseline data and results from 2 follow-up examinations. We divided the subjects into 3 groups according to baseline WTTR and used the Cox hazard regression model to estimate MAFLD risk.

**Results:**

The WTTRs were categorized by tertiles at baseline using the values 1.58 and 1.66. Patients with higher WTTR tended to have significantly greater values for the following factors, body mass index (BMI), fasting blood glucose (FPG), systolic blood pressure, diastolic blood pressure, total cholesterol (TC), triglycerides (TG), low-density lipoprotein-cholesterol (LDL-C) and neck circumference. The incidence of MAFLD significantly increased with increasing WTTR tertiles (5.74%, 12.75% and 22.25% for the first, second and third tertiles, respectively, *P* < 0.05 for trend). Kaplan-Meier(K-M) survival analysis revealed a significant tendency towards increased MAFLD risk with increasing WTTR tertile. In the fully adjusted model, the hazard ratios (95% CIs) for MAFLD in the second, third WTTR tertiles compared with the first quartile were 2.17(1.58,2.98), 3.63(2.70,4.89), respectively, third neck circumference tertiles compared with the first quartile were 2.84(1.89,4.25), 8.95(6.00,13.35), respectively. Compared with those of individuals with a BMI > 23 kg/m2, the associations between WTTR and MAFLD incidence were more pronounced in subjects with a BMI < 23 kg/m2. Similarly, the difference in neck circumference was more pronounced in these patients with a BMI < 23 kg/m2.

**Conclusions:**

Our results revealed that the WTTR is an independent risk factor for MAFLD, and there was a dose‒response relationship between the WTTR and MAFLD risk. The neck circumference was significantly different in subjects with a BMI < 23 kg/m2. This approach provides a new way to predict the incidence rate of MAFLD.

## Introduction

MAFLD is a new definition for nonalcoholic fatty liver disease (NAFLD) that was proposed by a panel of international experts [1].The prevalence of MAFLD continues to increase in tandem with global obesity rates [2], and MAFLD has become the world’s most common chronic liver disease [3]. In Chinese health care workers, MAFLD has also reached epidemic proportions, occurring in 37.4% of workers [4]. NAFLD, which includes a variety of clinicopathological entities, hepatic steatosis, hepatic steatohepatitis and hepatocellular carcinoma [5, 6], is believed to be a manifestation of metabolic syndrome in the liver [7]. MAFLD is often associated with obesity, insulin resistance, dyslipidaemia, and hypertension [6, 8]. MAFLD is diagnosed through ultrasound, which is the most common diagnostic method for MAFLD in clinical practice. Moreover, ultrasound examination is an easy to replicate and inexpensive technique, but there are many individual differences [8, 9]. At present, liver biopsy is the gold standard for diagnosing MAFLD [10]. However, this technique is invasive and inexpensive and has poor repeatability.

An increase in BMI is a risk factor for the occurrence of MAFLD [11], and a high BMI can significantly increase the burden of MAFLD [12]. According to the WHO criteria for Asians [13], subjects were classified as normal weight (BMI of 18.5–22.9 kg/m2) or overweight/obese (BMI ≥ 23 kg/m2). It has been reported that waist circumference is associated with the incidence of MAFLD [14]. The waist-to-thigh ratio has been identified as a significant predictor of diabetes and all-cause mortality [15, 16]. However, there are few reports indicating that the abdominal circumference to thigh circumference ratio is associated with the incidence of MAFLD. Based on the findings of a functional community queue of health care workers, this study explored the ratio of abdominal circumference to thigh circumference for the prediction of MAFLD.

## Materials and methods

### Subjects

This cohort was built using data from individuals who participated in a health check conducted by Peking university third hospital beginning on 1 July 2019. All subjects worked in the Peking University Third Hospital and underwent liver ultrasonography that was performed using the same equipment by the same experienced radiologist. MAFLD patients were diagnosed according to relevant guidelines and regulations [17], and MAFLD patients were selected based on abdominal ultrasonography. Patients were excluded if they had the following conditions: viral hepatitis; drug-induced hepatitis; excessive alcohol consumption; primary biliary cirrhosis; or severe liver, kidney, or thyroid dysfunction [4, 18]. Normal control individuals were selected based on abdominal ultrasonography, but those with liver disease were excluded. All patients underwent both a physical examination and a body composition analysis. This study is registered on China Clinical trials (http://www.chictr.org.cn/edit.aspx?pid=148121&htm=4).

#### Data collection, thigh circumference and abdominal circumference analysis

A physical examination, history, and body composition measurements were performed by a single trained health care provider. Subject histories included family history, drug history, smoking status, and alcohol intake. Abdominal ultrasound (HI VISION Ascendu, Japan) was routinely performed during a health check-up at our medical examination centre. The blood pressure of the subjects was measured by taking the average of two tests after they had rested for 5 min, and was subsequently measured by a trained nurse. Body composition was measured by bioelectrical impedance analysis (InBody770, Biospace Co.,Lid, Korea) [19], and thigh circumference, waist circumference and neck circumference were measured from the body composition [20].

#### Measurement of clinical parameters

BMI was calculated as weight in kilograms divided by height in metres squared (kg/m2). Body weight (kg) and height (m) were measured while the participants were in the standing position. FPG (enzymatic method), TC(enzymatic method), High density lipoprotein cholesterol (HDL-C) (enzymatic method), TG(enzymatic method), LDL-C(enzymatic method), alanine aminotransferase (ALT)(kinetic method), aspartate transaminase (AST) (kinetic method) were using an autoanalyzer (Cobas c 501 autoanalyzer, Roche Diagnostics, Germany). These materials can be detected through optical and electrochemical techniques (absorbance method, colorimetric method, fluorescence method, etc.) and can automatically complete sample loading, mixing, analysis, and result output.

### Statistical analysis

Spss26.0 was used for statistical analysis. The measurement data conforming to the normal distribution was expressed by means ± standard deviation, and independent sample t-test or were used for inter group comparison. The counting data were expressed by the number of cases (percentage), and the chi square test was used for the comparison between groups. We used Cox hazard regression models to assess hazard ratios (HRs) and 95% confidence intervals (CI) of incident MAFLD by WTTR tertiles for the time-dependent analyses. The waistline to thigh circumference Ratio were categorized by tertiles at baseline using the values 1.58 and 1.66. The incidence rate of MAFLD was calculated by R software (R4.3.2).

## Results

### Clinical characteristics

**Fig. 1:**
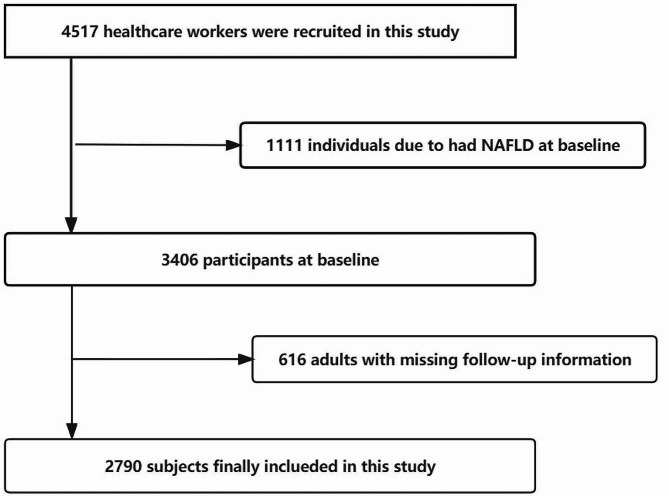
Flowchart for selection of study participants

A total of 4517 adults were recruited and invited to undergo a physical examination each year from 2019 to 2022. We excluded 1111 participants because they had MAFLD at baseline, and the remaining 3406 participants served as the baseline. Two follow-up visits were conducted in 2020 and 2021; 616 adults with missing follow-up information were excluded. Overall, 2790 subjects were included in this study (Fig. 1). The WTTRs were categorized by tertiles at baseline using the values 1.58 and 1.66. Patients with higher WTTR tended to have significantly greater values for the following factors: BMI (21.28 ± 2.39 vs. 22.40 ± 2.52 vs. 24.31 ± 2.90, *p* < 0.001), FPG(4.74 ± 0.42 vs. 4.86 ± 0.52 vs. 4.95 ± 0.62, *p* < 0.001), systolic pressure(113.57 ± 12.23 vs. 117.31 ± 13.45 vs. 121.54 ± 14.09, *p* < 0.001), diastolic pressure(69.14 ± 8.99 vs. 71.30 ± 9.32 vs. 73.55 ± 10.09, *p* < 0.001), TC(4.43 ± 0.77 vs. 4.56 ± 0.79 vs. 4.67 ± 0.84, *p* < 0.001), TG (0.75(0.59,0.98) vs. 0.86(0.65,1.13) vs. 1.03(0.76,1.40), *p* < 0.001), LDL-C(2.60 ± 0.68 vs. 2.77 ± 0.69 vs. 2.94 ± 0.73, *p* < 0.001) and neck circumference(32.19 ± 2.44 vs. 33.54 ± 2.32 vs. 33.56 ± 2.55, *p* < 0.001) (Table 1).

**Table 1:** Baseline characteristics of subjects by WTTR tertiles

Variables	T1(*n* = 1028)	T2(*n* = 863)	T3(*n* = 899)	*P*-Value
Age(Year) *	32.92 ± 7.61	35.25 ± 8.64	38.15 ± 10.09	< 0.001
Male(%)*	186(18.09)	174(20.16)	272(30.26)	< 0.001
Female(%)*	842(81.91)	689(79.84)	627(69.74)	< 0.001
BMI(kg/m^2^) *	21.28 ± 2.39	22.40 ± 2.52	24.31 ± 2.90	< 0.001
FPG(mmol/L) *	4.74 ± 0.42	4.86 ± 0.52	4.95 ± 0.62	< 0.001
ALT(U/L)*	13(10,17)	13(10,20)	16(12,22)	< 0.001
AST(U/L)*	17(15,20)	17(15,20)	18(16,21.5)	0.003
SBP(mmHg)*	113.57 ± 12.23	117.31 ± 13.45	121.54 ± 14.09	< 0.001
DBP(mmHg)*	69.14 ± 8.99	71.30 ± 9.32	73.55 ± 10.09	< 0.001
TC(mmol/L)*	4.43 ± 0.77	4.56 ± 0.79	4.67 ± 0.84	< 0.001
TG(mmol/L)*	0.75(0.59,0.98)	0.86(0.65,1.13)	1.03(0.76,1.40)	< 0.001
HDL-C(mmol/L)*	1.56 ± 0.31	1.49 ± 0.30	1.40 ± 0.30	< 0.001
LDL-C(mmol/L)*	2.60 ± 0.68	2.77 ± 0.69	2.94 ± 0.73	< 0.001
Neck circumference(cm)*	32.19 ± 2.44	33.54 ± 2.32	33.56 ± 2.55	< 0.001

Notes: Data are expressed as the mean ± standard deviation, M (Q_1_, Q_3_), n (%). Tertiles are based on baseline waistline to thigh circumference Ratio tertile; tertile 1, ≤ 1.58; tertile 2, 1.59–1.66; tertile 3, > 1.66. *P < 0.05 was considered statistically significant

The cumulative incidence of MAFLD was 13.2%, and the incidence of MAFLD was 61.40% per 1,000 person-years in health workers (95% CI 55.52–67.84). The following indicators, such as age, BMI, FPG, ALT, AST, SBP, DBP, TC, TG, LDL-C, HDL-C, the WTTR and neck circumference, were significantly different between the two groups (Table 2).

**Table 2:** The characteristics of MAFLD and control

Variables	Control (*n* = 2421)	MAFLD (*n* = 369)	*P*-Value
Age (Year)	34.96 ± 8.86	37.78 ± 9.85	< 0.001
Male (%)	477(19.70)	155(42.01)	< 0.001
Female (%)	1944(80.30)	214(57.99)	< 0.001
BMI (kg/m^2^)	22.22 ± 2.70	25.11 ± 2.89	< 0.001
FPG (mmol/L)	4.81 ± 0.47	5.05 ± 0.77	< 0.001
ALT(U/L)	13(10,19)	19(13,26)	< 0.001
AST(U/L)	17(15,20)	19(16,22)	< 0.001
SBP(mmHg)	116.41 ± 13.43	123.18 ± 13.50	< 0.001
DBP(mmHg)	70.70 ± 9.47	74.72 ± 9.91	< 0.001
TC(mmol/L)	4.52 ± 0.79	4.73 ± 0.88	< 0.001
TG(mmol/L)	0.82(0.63,1.09)	1.22(0.89,1.71)	< 0.001
HDL-C(mmol/L)	1.51 ± 0.31	1.31 ± 0.26	< 0.001
LDL-C(mmol/L)	2.72 ± 0.70	3.04 ± 0.75	< 0.001
WTTR	1.62 ± 0.09	1.68 ± 0.10	< 0.001
Neck circumference(cm)	33.36 ± 2.69	35.87 ± 2.59	< 0.001

Notes: *P* < 0.05 was considered statistically significant

**Fig. 2:**
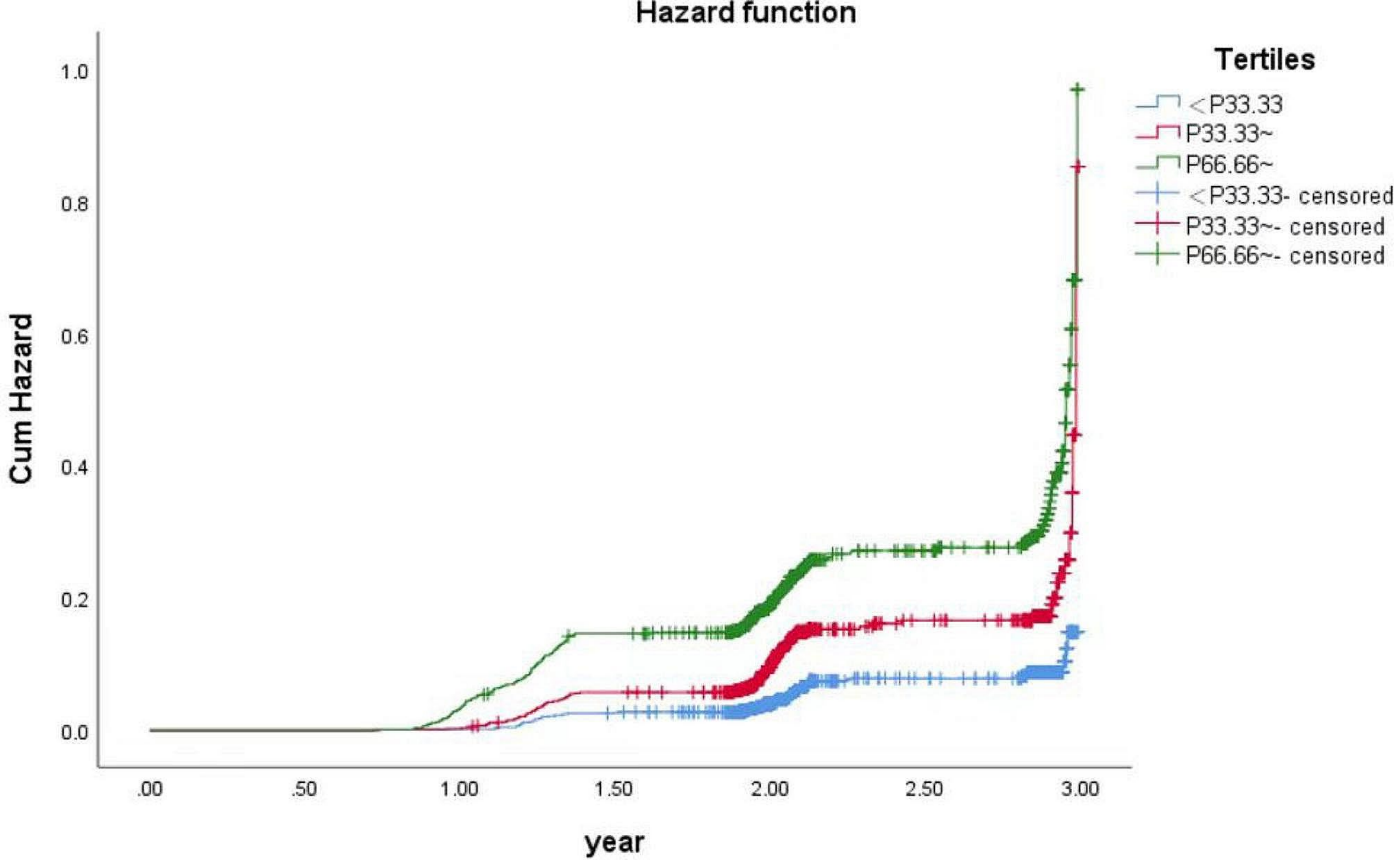
Kaplan-Meier curves for MAFLD by WTTR tertile

K-M survival analysis (Fig. 2) revealed a significant tendency towards increased MAFLD risk with increasing WTTR tertile. The incidence of MAFLD significantly increased with increasing WTTR tertiles (5.74%, 12.75% and 22.25% for the first, second and third tertiles, respectively; *P* < 0.05 for trend). Among the 3 groups, the first quartile had the lowest disease hazard for MAFLD, and the third tertile had the highest disease hazard. These findings indicated that higher WTTR levels predict greater incidences of MAFLD in a dose-dependent manner.

### The results of Cox proportional hazard regression

Cox proportional hazard regression was used to evaluate the association between WTTR tertiles or neck circumference and MAFLD development (Table 3). In the fully adjusted model, the hazard ratios (95% CIs) for MAFLD in the second, third WTTR tertiles compared with the first quartile were 2.17(1.58,2.98), 3.63(2.70,4.89), respectively (Table 4). Similarly, compared with those of the first quartile, the hazard ratios (95% CIs) for MAFLD in the second and third neck circumference tertiles were 2.84 (1.89,4.25) and 8.95 (6.00,13.35), respectively (Table 5).

**Table 3:** The characteristics of cox regression for MAFLD

Variables	HR	95%CI	*P*-Value
WTTR	1.035	1.024–1.047	< 0.001
FPG(mmol/L)	1.272	1.103–1.467	0.001
SBP(mmHg)	1.010	1.002–1.017	0.012
TG(mmol/L)	1.406	1.238–1.598	< 0.001
HDL-C(mmol/L)	0.346	0.222–0.538	< 0.001
LDL-C(mmol/L)	1.334	1.164–1.529	< 0.001
Neck circumference(cm)	1.270	1.210–1.334	< 0.001

Notes: *P* < 0.05 was considered statistically significant

**Table 4:** Cox proportional hazard regression result between WTTR level at baseline and incidence of MAFLD during follow-up

Variables	T1	T2	T3	*P*-value
Model1	1	2.28(1.66,3.13)	4.27(3.19,5.71)	< 0.001
Model2	1	2.17(1.58,2.98)	3.63(2.70,4.89)	< 0.001
Model3	1	1.82(1.229,2.55)	2.65(1.91,3.67)	< 0.001

Notes: Tertiles are based on abdomen- thigh ratio tertile: tertile 1, ≤ 1.58; tertile 2, 1.59–1.66; tertile 3, > 1.66. *P* < 0.05 was considered statistically significant. Model 1: unadjusted baseline values of variables;

model 2: adjusted for gender and age; model 3: adjusted for gender, age, FPG, SBP, DBP, TG, HDL-C, LDL-C.

**Table 5:** Cox proportional hazard regression result between neck circumference at baseline and incidence of MAFLD during follow-up

Variables	T1	T2	T3	*P*-value
Model1	1	2.91(1.94,4.35)	8.85(6.12,12.79)	< 0.001
Model2	1	2.84(1.89,4.25)	8.95(6.00,13.35)	< 0.001
Model3	1	2.27(1.49,3.47)	5.19(3.37,7.99)	< 0.001

Tertiles are based on baseline neck circumference tertile: tertile 1, ≤ 32.1; tertile 2, 32.2–34.6; tertile 3, > 34.7. Model 1: unadjusted baseline values of variables; model 2: adjusted for gender and age;

model 3: adjusted for gender, age, FPG, SBP, DBP, TG, HDL-C, LDL-C.

### Subgroup analysis of incident MAFLD

Subgroup analysis was also conducted to determine the HRs and CIs for incident MAFLD according to BMI (< 23, ≥ 23 kg/m2). Table 6 shows that the association between WTTR and the incidence of MAFLD was more pronounced in subjects with a BMI < 23 kg/m2. Similarly, neck circumference was more pronounced in subjects with a BMI < 23 kg/m2, but not in those with a BMI ≥ 23 kg/m2 (Table 7).

**Table 6:** Cox proportional hazard regression of WTTR for subgroup analysis

Subgroup	N		T1	T2	T3	*P*-value
BMI ≥ 23 kg/m2	1161	Model1	1	1.49(1.02,2.19)	1.81(1.28,2.57)	<0.05
		Model2	1	1.46(0.99,2.15)	1.78(1.25,2.53)	<0.05
		Model3	1	1.29(0.85,1.96)	1.56(1.07,2.29)	<0.05
BMI<23 kg/m2	1629	Model1	1	2.29(1.31,4.02)	4.29(2.44,7.56)	< 0.001
		Model2	1	2.31(1.31,4.05)	3.89(2.17,6.97)	< 0.001
		Model3	1	1.87(1.03,3.40)	2.57(1.36,4.85)	< 0.001

Notes: Tertiles are based on abdomen- thigh ratio tertile: tertile 1, ≤ 1.58; tertile 2, 1.59–1.66; tertile 3, > 1.66. *P* < 0.05 was considered statistically significant. Model 1: unadjusted baseline values of variables;

model 2: adjusted for gender and age; model 3: adjusted for gender, age, FPG, SBP, DBP, TG, HDL-C, LDL-C.

**Table 7:** Cox proportional hazard regression of neck circumference for subgroup analysis

Subgroup	N		T1	T2	T3	*P*-value
BMI ≥ 23 kg/m2	1161	Model1	1	0.93(0.42,2.05)	2.14(1.00,4.57)	0.858
		Model2	1	0.92(0.42,2.04)	1.91(0.88,4.14)	0.839
		Model3	1	0.93(0.40,2.20)	1.63(0.70,3.79)	0.874
BMI<23 kg/m2	1629	Model1	1	2.22(1.32,3.74)	4.56(2.59,8.03)	0.003
		Model2	1	2.24(1.32,3.78)	6.38(2.42,16.81)	0.003
		Model3	1	1.65(0.95,2.86)	3.25(1.16,9.14)	0.074

Tertiles are based on baseline neck circumference tertile: tertile 1, ≤ 32.1; tertile 2, 32.2–34.6; tertile 3, > 34.7. Model 1: unadjusted baseline values of variables; model 2: adjusted for gender and age; model 3: adjusted for gender, age, FPG, SBP, DBP, TG, HDL-C, LDL-C.

## Discussion

Our results showed that subjects with higher WTTRs tended to have significantly greater levels of the following factors: BMI, FPG, blood pressure, TC, TG and LDL-C. A study in the United States reported that waist circumference is a risk factor for MAFLD [21]. Moreover, a previous report revealed a greater risk of diabetes in U.S. men with increased waist circumference [22], and a Filipino study reported that an increase in waist circumference is positively correlated with the risk of hypertension [23]. Other studies have shown that an increase in waist circumference leads to an increased incidence of hyperlipidaemia [24, 25]. These findings are consistent with our research results. The incidence of 46.13 new MAFLD cases per 1,000 person-years has been reported on a global scale [26]. Since 2015, the incidence of MAFLD in China has continued to increase, and 82.59 cases per 1,000 person-years have been reported [27]. Similarly, our study revealed that the incidence of MAFLD was 61.4% of cases per 1,000 person-years among health workers, which is consistent with the findings of the literature above. Moreover, we found that neck circumference is associated with the incidence of MAFLD. Previous literature has shown that neck circumference could be used as a simple predictive tool for NAFLD [28]. Second, our results indicated that higher WTTR predicted a greater incidence of MAFLD in a dose-dependent manner. A previous study revealed that the WTTR was the best indicator of type 2 diabetes [29]. There is a greater level of WTTR in obese objects than in nonobese individuals [30]. Similarly, increased waist circumference is the main reason for the rapid increase in the incidence of MAFLD [14]. Previous literature has reported that an increase in the ratio of thigh circumference to waist circumference reduces the risk of MAFLD [31, 32]. It is reported that low femoral subcutaneous fat amounts were shown to be independently associated with fatty liver disease [33], and a low leg fat to total fat ratio remained a risk factor for MAFLD [34]. Therefore, we speculate that WTTR is associated with MAFLD. Third, this study showed that the association between WTTR and the incidence of MAFLD was more pronounced in subjects with a BMI < 23 kg/m2, and neck circumference was significantly different in subjects with a BMI < 23 kg/m2. Similarly, it has been reported that the impact of a waist circumference increase is greater for individuals with a BMI < 25 kg/m2 than for those with a BMI ≥ 25 kg/m2 [35], which further supports our research findings. This may be because fat distribution has a greater impact on MAFLD [31, 36]. Furthermore, some studies have reported uneven fat distribution, indicating that the ability to increase the size and number of adipocytes in subcutaneous tissue is ultimately limited. In this case, lipids accumulate in other less adapted tissues, especially the liver [36, 37]. Additionally, uneven fat distribution may be associated with insulin resistance [38, 39]. Insulin resistance is associated with the occurrence of MAFLD [40]. Another factor in these adults is work-related stress [41–43], such as night shiftwork, which can increase the risk of MAFLD.

### Limitations

Our results should be considered in the context of several limitations. First, we analysed only health care workers, and we will further study other occupational groups in the future. Second, there are several limitations in terms of sample representativeness for single-centre surveys, and multicentre research should be conducted in the future. Third, we speculate that the underlying mechanism is related to insulin resistance, fat distribution, and work-related stress-related mechanisms. In the future, we will further study the underlying mechanism involved, and we will collect additional glycated haemoglobin data or other biochemical data in the future. Additionally, we will further evaluate the relationships between other indicators and MAFLD. Moreover, we further analysed the relationships between lifestyle and MAFLD among health care workers, such as sedentaryism and diet patterns.

## Conclusions

Our results indicated that a greater ratio of abdominal circumference to thigh circumference is associated with an increased incidence of MAFLD in health care workers. Compared with those in patients with a BMI ≥ 23 kg/m2, the WTTR and MAFLD incidence were significantly greater in patients with a BMI < 23 kg/m2, and the neck circumference was significantly different between these two groups. This finding provides new ideas for the occurrence of MAFLD in the future.

## Data Availability

The original contributions presented in the study are included in the article material, further inquiries can be directed to the corresponding author.

## Abbreviations

WTTR: waist-to-thigh ratio
MAFLD: metabolic associated fatty liver disease
NAFLD: nonalcoholic fatty liver disease
BMI: body mass Index
FPG: fasting blood glucose
TC: total cholesterol
TG: triglycerides
LDL-C: low-density lipoprotein-cholesterol
HDL-C: high density lipoprotein cholesterol
ALT: alanine aminotransferase
AST: aspartate transaminase
CI: confidence intervals
HRs: hazard ratios
